# Supplementary material for: Randomness analysis of end-to-end delay in random forwarding networks
Source: PeerJ Comput Sci. 2022 Apr 6;8:e942. doi: 10.7717/peerj-cs.942 (PMC9044350; doi:10.7717/peerj-cs.942)
Supplement: Supplemental Information 1 [file peerj-cs-08-942-s001.pdf]

1 # APPENDIX

2 The appendix mainly proves the rotation consistency of symbol matrix.

Generally, let the symmetric symbol matrix $\boldsymbol{S}_m$ be

$$\boldsymbol{S}_m = \begin{pmatrix} 1 & x_1 & x_2 & \dots & x_{m-1} \\ x_1 & 1 & x_m & x_{m+1} & \dots \\ x_2 & x_m & 1 & \dots & \dots \\ \dots & x_{m+1} & \dots & 1 & x_n \\ x_{m-1} & \dots & \dots & x_n & 1 \end{pmatrix}$$

3 ## cyclic permutation transformation $\boldsymbol{R}$

First, the cyclic permutation transformation R is defined as

$$\begin{pmatrix} f_1(\boldsymbol{X}) \\ f_2(\boldsymbol{X}) \\ \dots \\ f_m(\boldsymbol{X}) \end{pmatrix} \xrightarrow{R} \begin{pmatrix} f_m(\sigma(\boldsymbol{X})) \\ f_1(\sigma(\boldsymbol{X})) \\ \dots \\ f_{m-1}(\sigma(\boldsymbol{X})) \end{pmatrix}$$

where $\boldsymbol{X} = (x_1, x_2, \dots, x_n)$ and permutation operator $\boldsymbol{\sigma} = \begin{pmatrix} x_1 & x_2 & \dots & x_n \\ \sigma(x_1) & \sigma(x_2) & \dots & \sigma(x_n) \end{pmatrix}$ and it makes

$$\boldsymbol{S}_m = \begin{pmatrix} \boldsymbol{\mu} & R(\boldsymbol{\mu}) & \dots & R^{m-1}(\boldsymbol{\mu}) \end{pmatrix}, \quad \boldsymbol{\mu} = \begin{pmatrix} 1 & x_1 & x_2 & \dots & x_{m-1} \end{pmatrix}^T$$

4 Operator R has the following two important properties:

- **Property 1**

$$R^m(\boldsymbol{\alpha}) = \boldsymbol{\alpha}, \quad \boldsymbol{\alpha} \in \mathbb{R}^m$$

5 **Proof**

6 Let $\boldsymbol{S}_m = \begin{pmatrix} \boldsymbol{\mu} & R(\boldsymbol{\mu}) & \dots & R^{m-1}(\boldsymbol{\mu}) \end{pmatrix}, \boldsymbol{\mu} = \begin{pmatrix} 1 & x_1 & x_2 & \dots & x_{m-1} \end{pmatrix}^T$.

Since $\boldsymbol{S}_m = \boldsymbol{S}_m^T$, the m-column vector is equal to the m-row vector of $\boldsymbol{S}_m$, that is

$$R^{m-1}(\boldsymbol{\mu}) = \begin{pmatrix} x_{m-1} \\ \dots \\ \dots \\ x_n \\ 1 \end{pmatrix} = \begin{pmatrix} \boldsymbol{\mu}_m \\ R(\boldsymbol{\mu})_m \\ \dots \\ R^{m-2}(\boldsymbol{\mu})_m \\ 1 \end{pmatrix}$$

$$R^m(\boldsymbol{\mu}) = R(R^{m-1}(\boldsymbol{\mu})) = R(\begin{pmatrix} \boldsymbol{\mu}_m \\ R(\boldsymbol{\mu})_m \\ \dots \\ R^{m-2}(\boldsymbol{\mu})_1 \\ 1 \end{pmatrix}) = \begin{pmatrix} 1 \\ R(\boldsymbol{\mu})_1 \\ \dots \\ R^{m-1}(\boldsymbol{\mu})_1 \\ R^m(\boldsymbol{\mu})_1 \end{pmatrix} = \begin{pmatrix} 1 \\ x_1 \\ x_2 \\ \dots \\ x_{m-1} \end{pmatrix} = \boldsymbol{\mu}$$

7 where $\boldsymbol{\mu}_m$ denotes the mth element of $\boldsymbol{\mu}$, and so on.

So, $\sigma^m(\boldsymbol{X}) = \boldsymbol{X}$. Then for $\boldsymbol{\alpha} = \begin{pmatrix} f_1(\boldsymbol{X}) \\ f_2(\boldsymbol{X}) \\ \dots \\ f_m(\boldsymbol{X}) \end{pmatrix}$, we have

$$R^m(\boldsymbol{\alpha}) = \begin{pmatrix} f_1(\sigma^m(\boldsymbol{X})) \\ f_2(\sigma^m(\boldsymbol{X})) \\ \dots \\ f_m(\sigma^m(\boldsymbol{X})) \end{pmatrix} = \begin{pmatrix} f_1(\boldsymbol{X}) \\ f_2(\boldsymbol{X}) \\ \dots \\ f_m(\boldsymbol{X}) \end{pmatrix} = \boldsymbol{\alpha}$$

- **Property 2**

$$R(\boldsymbol{\alpha})^T R(\boldsymbol{\beta}) = \sigma(\boldsymbol{\alpha}^T \boldsymbol{\beta}), \quad \boldsymbol{\alpha}, \boldsymbol{\beta} \in \mathbb{R}^m$$

8 **Proof**

Suppose $\boldsymbol{\alpha} = \begin{pmatrix} f_1(\boldsymbol{X}) \\ f_2(\boldsymbol{X}) \\ \dots \\ f_m(\boldsymbol{X}) \end{pmatrix}, \boldsymbol{\beta} = \begin{pmatrix} g_1(\boldsymbol{X}) \\ g_2(\boldsymbol{X}) \\ \dots \\ g_m(\boldsymbol{X}) \end{pmatrix}$, then

$$\sigma(\boldsymbol{\alpha}^T\boldsymbol{\beta}) = \sigma(\sum_{i=1}^{m} f_i(\boldsymbol{X})g_i(\boldsymbol{X})) = \sum_{i=1}^{m} f_i(\sigma(\boldsymbol{X}))g_i(\sigma(\boldsymbol{X}))$$

While

$$R(\boldsymbol{\alpha})^T R(\boldsymbol{\beta}) = \begin{pmatrix} f_m(\sigma(\boldsymbol{X})) \\ f_1(\sigma(\boldsymbol{X})) \\ \dots \\ f_{m-1}(\sigma(\boldsymbol{X})) \end{pmatrix}^T \begin{pmatrix} g_m(\sigma(\boldsymbol{X})) \\ g_1(\sigma(\boldsymbol{X})) \\ \dots \\ g_{m-1}(\sigma(\boldsymbol{X})) \end{pmatrix} = \sum_{i=1}^{m} f_i(\sigma(\boldsymbol{X}))g_i(\sigma(\boldsymbol{X}))$$

So

$$R(\boldsymbol{\alpha})^T R(\boldsymbol{\beta}) = \sigma(\boldsymbol{\alpha}^T\boldsymbol{\beta})$$

Next, we will prove that matrix multiplication is ***R*-preserving** operation.

Proof

Suppose matrix $\boldsymbol{A} = \begin{pmatrix} \boldsymbol{\alpha} & R(\boldsymbol{\alpha}) & \dots & R^{m-1}(\boldsymbol{\alpha}) \end{pmatrix}$ and matrix $\boldsymbol{B} = \begin{pmatrix} \boldsymbol{\beta} & R(\boldsymbol{\beta}) & \dots & R^{m-1}(\boldsymbol{\beta}) \end{pmatrix}$. Then the product of the two is

$$\boldsymbol{AB} = \boldsymbol{A}^T\boldsymbol{B} = \begin{pmatrix} \boldsymbol{\alpha}^T \\ R(\boldsymbol{\alpha})^T \\ \dots \\ R^{m-1}(\boldsymbol{\alpha})^T \end{pmatrix} \begin{pmatrix} \boldsymbol{\beta} & R(\boldsymbol{\beta}) & \dots & R^{m-1}(\boldsymbol{\beta}) \end{pmatrix}$$

$$= \begin{pmatrix} \boldsymbol{\alpha}^T\boldsymbol{\beta} & \boldsymbol{\alpha}^T R(\boldsymbol{\beta}) & \dots & \boldsymbol{\alpha}^T R^{m-1}(\boldsymbol{\beta}) \\ R(\boldsymbol{\alpha})^T\boldsymbol{\beta} & R(\boldsymbol{\alpha})^T R(\boldsymbol{\beta}) & \dots & R(\boldsymbol{\alpha})^T R^{m-1}(\boldsymbol{\beta}) \\ \dots & \dots & \dots & \dots \\ R^{m-1}(\boldsymbol{\alpha})^T\boldsymbol{\beta} & R^{m-1}(\boldsymbol{\alpha})^T R(\boldsymbol{\beta}) & \dots & R^{m-1}(\boldsymbol{\alpha})^T R^{m-1}(\boldsymbol{\beta}) \end{pmatrix}$$

Let $\boldsymbol{\gamma_i}$ denotes the i-th column of $\boldsymbol{AB}$, that is

$$\boldsymbol{\gamma_i} = \begin{pmatrix} \boldsymbol{\alpha}^T R^{i-1}(\boldsymbol{\beta}) \\ R(\boldsymbol{\alpha})^T R^{i-1}(\boldsymbol{\beta}) \\ \dots \\ R^{m-1}(\boldsymbol{\alpha})^T R^{i-1}(\boldsymbol{\beta}) \end{pmatrix}$$

Then

$$R(\boldsymbol{\gamma_i}) = R\begin{pmatrix} \boldsymbol{\alpha}^T R^{i-1}(\boldsymbol{\beta}) \\ R(\boldsymbol{\alpha})^T R^{i-1}(\boldsymbol{\beta}) \\ \dots \\ R^{m-1}(\boldsymbol{\alpha})^T R^{i-1}(\boldsymbol{\beta}) \end{pmatrix} = \begin{pmatrix} \sigma(R^{m-1}(\boldsymbol{\alpha})^T R^{i-1}(\boldsymbol{\beta})) \\ \sigma(\boldsymbol{\alpha}^T R^{i-1}(\boldsymbol{\beta})) \\ \dots \\ \sigma(R^{m-2}(\boldsymbol{\alpha})^T R^{i-1}(\boldsymbol{\beta})) \end{pmatrix}$$

$$= \begin{pmatrix} R^{m}(\boldsymbol{\alpha})^T R^{i}(\boldsymbol{\beta}) \\ R(\boldsymbol{\alpha})^T R^{i}(\boldsymbol{\beta}) \\ \dots \\ R^{m-1}(\boldsymbol{\alpha})^T R^{i}(\boldsymbol{\beta}) \end{pmatrix} = \begin{pmatrix} \boldsymbol{\alpha}^T R^{i}(\boldsymbol{\beta}) \\ R(\boldsymbol{\alpha})^T R^{i}(\boldsymbol{\beta}) \\ \dots \\ R^{m-1}(\boldsymbol{\alpha})^T R^{i}(\boldsymbol{\beta}) \end{pmatrix} = \boldsymbol{\gamma_{i+1}}$$

So

$$\boldsymbol{AB} = \begin{pmatrix} \boldsymbol{\gamma_1} & R(\boldsymbol{\gamma_1}) & \dots & R^{m-1}(\boldsymbol{\gamma_1}) \end{pmatrix}$$

That is to say, matrix multiplication is ***R*-preserving** operation. Then we can assert that $\boldsymbol{S}_m^N$ also has the cyclic permutation characteristics. Let $\boldsymbol{\mu_i}$ denotes the first column of $\boldsymbol{S}_m^i$, then $\boldsymbol{S}_m^N$ can be rewritten as

$$\boldsymbol{S}_m^N = \begin{pmatrix} \boldsymbol{\mu_N} & R(\boldsymbol{\mu_N}) & \dots & R^{m-1}(\boldsymbol{\mu_N}) \end{pmatrix}$$

This ensures that only $\boldsymbol{\mu_N}$ needs to be known, and the whole matrix $\boldsymbol{S}_m^N$ can be recovered by continuously carrying out the cyclic permutation transformation R, and R also ensures that $\boldsymbol{S}_m^N$ has rotation consistency between rows and columns.

15 **replacement transformation e_{ij}**

Second, the rotation consistency within the rows and columns of the symbol matrix is guaranteed by the replacement transformation e_{ij}

$$f_i(\boldsymbol{X}) \overset{e_{ij}}{\to} f_j(\boldsymbol{X}) = f_i(e_{ij}(\boldsymbol{X})), \quad i, j \geq 2$$

The replacement rule is that the symbols corresponding to the i-th column and the j-th column of S_m are exchanged with each other. Take S_4 as an example, the second column of $\boldsymbol{S}_4$ is $\begin{pmatrix} x_1 & 1 & x_3 & x_4 \end{pmatrix}^T$ and the third column of $\boldsymbol{S}_4$ is $\begin{pmatrix} x_2 & x_3 & 1 & x_5 \end{pmatrix}^T$, then the exchange rule is

$$e_{23} = \begin{cases} x_1 \leftrightarrow x_2 \\ x_4 \leftrightarrow x_5 \end{cases}$$

16 In which, $1 \leftrightarrow x_3$ and $x_3 \leftrightarrow 1$ are mutually inverse and cancelled.

Then $\boldsymbol{\mu}$ can be expressed by e_{ij} as

$$\boldsymbol{\mu} = \begin{pmatrix} f_1(\boldsymbol{X}) \\ f_2(\boldsymbol{X}) \\ f_3(\boldsymbol{X}) \\ \cdots \\ f_m(\boldsymbol{X}) \end{pmatrix} = \begin{pmatrix} f_1(\boldsymbol{X}) \\ f_2(\boldsymbol{X}) \\ f_2(e_{23}(\boldsymbol{X})) \\ \cdots \\ f_m(e_{2m}(\boldsymbol{X})) \end{pmatrix}$$

17 Operator e_{ij} has the following four important properties:

- **Property 1**

$$e_{ij} = e_{ij}^{-1} = e_{ji}$$

18 **Proof**

From the definition of e_{ij}: $f_i(\boldsymbol{X}) \overset{e_{ij}}{\to} f_j(\boldsymbol{X})$, we have

$$f_j(\boldsymbol{X}) \overset{e_{ij}^{-1}}{\to} f_i(\boldsymbol{X})$$

So

$$e_{ij}^{-1} = e_{ji}$$

And it is defined as the symbol exchange between the i-th column and the j-th column of S_m, so

$$e_{ij} = e_{ji}$$

Therefore

$$e_{ij} = e_{ij}^{-1} = e_{ji}$$

- **Property 2**

$$e_{ij}^2 = I$$

19 where I denotes identical transformation, that is $I(\boldsymbol{X}) = \boldsymbol{X}$

20 **Proof**

According to property 1, we know $e_{ij} = e_{ij}^{-1}$, so

$$e_{ij}^2 = e_{ij} e_{ij}^{-1} = I$$

- **Property 3**

$$f_1(e_{ij}(\boldsymbol{X})) = f_1(\boldsymbol{X})$$

21 **Proof**

22 Suppose $\boldsymbol{S}_m = \begin{pmatrix} \boldsymbol{\mu_1} & R(\boldsymbol{\mu_1}) & \dots & R^{m-1}(\boldsymbol{\mu_1}) \end{pmatrix}$, $\boldsymbol{\mu_1} = \begin{pmatrix} 1 & x_1 & x_2 & \dots & x_{m-1} \end{pmatrix}^T$.

Because $\boldsymbol{S}_m^N = \boldsymbol{S}_m^{N-1} \boldsymbol{S}_m$, for the first column $\boldsymbol{\mu_N}$ of $\boldsymbol{S}_m^N$, we have

$$\boldsymbol{\mu_N} = \boldsymbol{S}_m^N \boldsymbol{\mu_1}$$

Then for the first element $f_1(\boldsymbol{X})$ of $\boldsymbol{\mu_1}$, we have

$$f_1(\boldsymbol{X}) = \boldsymbol{\mu_{N-1}}^T\boldsymbol{\mu_1} = (\boldsymbol{S}_m^{N-2}\boldsymbol{\mu_1})^T\boldsymbol{\mu_1} = \boldsymbol{\mu_1}^T\boldsymbol{S}_m^{N-2}\boldsymbol{\mu_1}$$

So

$$f_1(e_{ij}(\boldsymbol{X})) = e_{ij}(\boldsymbol{\mu_1})^T e_{ij}(\boldsymbol{S}_m)^{N-2} e_{ij}(\boldsymbol{\mu_1}) = \pi_{ij}(\boldsymbol{\mu_1})^T(\boldsymbol{P}_{ij}\boldsymbol{S}_m\boldsymbol{P}_{ij})^{N-2}\pi_{ij}(\boldsymbol{\mu_1})$$
$$= \pi_{ij}(\boldsymbol{\mu_1})^T(\boldsymbol{P}_{ij}\boldsymbol{S}_m^{N-2}\boldsymbol{P}_{ij})\pi_{ij}(\boldsymbol{\mu_1}) = \boldsymbol{\mu_1}^T\boldsymbol{S}_m^{N-2}\boldsymbol{\mu_1} = f_1(\boldsymbol{X})$$

where π_{ij} is the exchange operator to exchange the i-th element and the j-th element of a vector. $\boldsymbol{P}_{ij}$ is the permutation matrix. The left multiplication by $\boldsymbol{P}_{ij}$ exchanges the i-th and j-th rows of a matrix, and the right multiplication by $\boldsymbol{P}_{ij}$ exchanges the i-th and j-th columns of a matrix.

- **Property 4**

$$f_i(e_{2j}(\boldsymbol{X})) = f_i(\boldsymbol{X}), \quad i \neq j, \quad i,j > 2$$

Proof

It is proved by mathematical induction.

1. For $\boldsymbol{S}_m = \begin{pmatrix}\boldsymbol{\mu_1} & R(\boldsymbol{\mu_1}) & \dots & R^{m-1}(\boldsymbol{\mu_1})\end{pmatrix}$, we have

$$\boldsymbol{\mu_1} = \begin{pmatrix}f_1(\boldsymbol{X}) & f_2(\boldsymbol{X}) & f_3(\boldsymbol{X}) & \dots & f_m(\boldsymbol{X})\end{pmatrix}^T = \begin{pmatrix}1 & x_1 & x_2 & \dots & x_{m-1}\end{pmatrix}^T$$

According to the definition of e_{ij}, e_{2j} exchanges the second column and the j-th column. While $f_i(\boldsymbol{X})$ is in the first row and the i-th column and $i \neq j$, $i,j > 2$, e_{2j} has no effect on the first row and the i-th column of $\boldsymbol{S}_m$. So for $\boldsymbol{S}_m$, $f_i(e_{2j}(\boldsymbol{X})) = f_i(\boldsymbol{X})$.

2. Suppose the first column $\boldsymbol{\mu_N} = \begin{pmatrix}f_1(\boldsymbol{X}) & f_2(\boldsymbol{X}) & f_3(\boldsymbol{X}) & \dots & f_{m-1}(\boldsymbol{X})\end{pmatrix}^T$ of $\boldsymbol{S}_m^N$ satisfies $f_i(e_{2j}(\boldsymbol{X})) = f_i(\boldsymbol{X})$, $i \neq j$, $i,j > 2$.
Then for the first column of $\boldsymbol{S}_m^{N+1} = \boldsymbol{S}_m\boldsymbol{S}_m^N$, we have

$$\boldsymbol{\mu_{N+1}} = \boldsymbol{S}_m\boldsymbol{\mu_N}$$

Let $\boldsymbol{\mu_{N+1}} = \begin{pmatrix}f_1^{'}(\boldsymbol{X}) & f_2^{'}(\boldsymbol{X}) & f_3^{'}(\boldsymbol{X}) & \dots & f_{m-1}^{'}(\boldsymbol{X})\end{pmatrix}^T$, we have

$$f_i^{'}(\boldsymbol{X}) = R^{i-1}(\boldsymbol{\mu_1})^T\boldsymbol{\mu_N}$$

$$f_i^{'}(e_{2j}(\boldsymbol{X})) = e_{2j}(R^{i-1}(\boldsymbol{\mu_1}))^T e_{2j}(\boldsymbol{\mu_N})$$

where $R^{i-1}(\boldsymbol{\mu_1})$ is the i-th column of $\boldsymbol{S}_m$ and is also j-th column of $\boldsymbol{S}_m$ because of the symmetry of $\boldsymbol{S}_m$. Because $i \neq j$, $i,j > 2$, e_{2j} only exchange the second element and the j-th element of $R^{i-1}(\boldsymbol{\mu_1})$, we have

$$e_{2j}(R^{i-1}(\boldsymbol{\mu_1})) = \pi_{2j}(R^{i-1}(\boldsymbol{\mu_1}))$$

While $e_{2j}(\boldsymbol{\mu_N}) = \begin{pmatrix}f_1(e_{2j}(\boldsymbol{X})) & f_2(e_{2j}(\boldsymbol{X})) & f_3(e_{2j}(\boldsymbol{X})) & \dots & f_{m-1}(e_{2j}(\boldsymbol{X}))\end{pmatrix}^T$, according to property 3, we know $f_1(e_{2j}(\boldsymbol{X})) = f_1(\boldsymbol{X})$. And because $f_2(e_{2j}(\boldsymbol{X})) = f_j(\boldsymbol{X})$, $f_j(e_{2j}(\boldsymbol{X})) = f_j(e_{j2}(\boldsymbol{X})) = f_2(\boldsymbol{X})$, for other $f_i(e_{2j}(\boldsymbol{X}))$, $i \neq j$, $i,j > 2$, according to the hypothesis, we have $f_i(e_{2j}(\boldsymbol{X})) = f_i(\boldsymbol{X})$, so

$$e_{2j}(\boldsymbol{\mu_N}) = \pi_{2j}(\boldsymbol{\mu_N})$$

Therefore

$$f_i^{'}(e_{2j}(\boldsymbol{X})) = \pi_{2j}(R^{i-1}(\boldsymbol{\mu_1}))^T\pi_{2j}(\boldsymbol{\mu_N}) = R^{i-1}(\boldsymbol{\mu_1})^T\boldsymbol{\mu_N} = f_i^{'}(\boldsymbol{X})$$

3. In conclusion, $f_i(e_{2j}(\boldsymbol{X})) = f_i(\boldsymbol{X})$, $i \neq j$, $i,j > 2$.

Similarly, we will prove that matrix multiplication is **$\boldsymbol{e_{ij}}$-preserving** operation.

Proof

Suppose $\boldsymbol{S}_m = \begin{pmatrix}\boldsymbol{\mu_1} & R(\boldsymbol{\mu_1}) & \dots & R^{m-1}(\boldsymbol{\mu_1})\end{pmatrix}$, $\boldsymbol{\mu_1} = \begin{pmatrix}1 & x_1 & x_2 & \dots & x_{m-1}\end{pmatrix}^T$. It is also proved by mathematical induction.

1. For the first column $\boldsymbol{\mu_1}$ of $\boldsymbol{S}_m$, we have

$$\boldsymbol{\mu_1} = \begin{pmatrix} f_1(\boldsymbol{X}) & f_2(\boldsymbol{X}) & f_3(\boldsymbol{X}) & \dots & f_m(\boldsymbol{X}) \end{pmatrix}^T = \begin{pmatrix} 1 & x_1 & x_2 & \dots & x_{m-1} \end{pmatrix}^T$$

   According to the definition of e_{ij}, it is obviously that $f_j(\boldsymbol{X}) = f_i(e_{ij}(\boldsymbol{X})),\ i,j \geq 2$.

2. Suppose the first column $\boldsymbol{\mu}_N = \begin{pmatrix} f_1(\boldsymbol{X}) & f_2(\boldsymbol{X}) & f_3(\boldsymbol{X}) & \dots & f_{m-1}(\boldsymbol{X}) \end{pmatrix}^T$ of $\boldsymbol{S}_m^N$ satisfies $f_j(\boldsymbol{X}) = f_i(e_{ij}(\boldsymbol{X})),\ i,j \geq 2$.
   Then for the first column of $\boldsymbol{S}_m^{N+1} = \boldsymbol{S}_m \boldsymbol{S}_m^N$, we have

$$\boldsymbol{\mu_{N+1}} = \boldsymbol{S}_m \boldsymbol{\mu_N}$$

   Let $\boldsymbol{\mu_{N+1}} = \begin{pmatrix} f_1'(\boldsymbol{X}) & f_2'(\boldsymbol{X}) & f_3'(\boldsymbol{X}) & \dots & f_{m-1}'(\boldsymbol{X}) \end{pmatrix}^T$, we have

$$f_j'(\boldsymbol{X}) = R^{j-1}(\boldsymbol{\mu_1})^T \boldsymbol{\mu_N} = \pi_{ij}(R^{j-1}(\boldsymbol{\mu_1}))^T \pi_{ij}(\boldsymbol{\mu_N})$$

   According to the properties of e_{ij} and the hypothesis, we have

$$\pi_{ij}(\boldsymbol{\mu_N}) = \begin{pmatrix} f_1(\boldsymbol{X}) \\ \dots \\ f_j(\boldsymbol{X}) \\ \dots \\ f_i(\boldsymbol{X}) \\ \dots \\ f_m(\boldsymbol{X}) \end{pmatrix} = \begin{pmatrix} f_1(e_{ij}(\boldsymbol{X})) \\ \dots \\ f_i(e_{ij}(\boldsymbol{X})) \\ \dots \\ f_j(e_{ij}(\boldsymbol{X})) \\ \dots \\ f_m(e_{ij}(\boldsymbol{X})) \end{pmatrix} = e_{ij}(\boldsymbol{\mu_N}), \quad i,j > 2$$

   And because $e_{ij}(R^{i-1}(\boldsymbol{\mu_1})) = \pi_{ij}(R^{j-1}(\boldsymbol{\mu_1}))$, then

$$f_j'(\boldsymbol{X}) = \pi_{ij}(R^{j-1}(\boldsymbol{\mu_1}))^T \pi_{ij}(\boldsymbol{\mu_N}) = e_{ij}(R^{i-1}(\boldsymbol{\mu_1}))^T e_{ij}(\boldsymbol{\mu_N})$$

$$= e_{ij}(R^{i-1}(\boldsymbol{\mu_1})\boldsymbol{\mu_N}) = e_{ij}(f_i'(\boldsymbol{X})) = f_i'(e_{ij}(\boldsymbol{X})), \quad i,j > 2$$

3. In conclusion, $f_j(\boldsymbol{X}) = f_i(e_{ij}(\boldsymbol{X})),\ i,j \geq 2$ is always true for any $\boldsymbol{S}_m^N$.

Finally, because matrix multiplication is **$\boldsymbol{R}$-preserving** operation and **$\boldsymbol{e_{ij}}$-preserving** operation, the rotation consistency of symbol matrix is true for any number of nodes and any number of forwarding times.
